# Intercultural Psychotherapy - needs and challenges

**DOI:** 10.1192/j.eurpsy.2025.50

**Published:** 2025-08-26

**Authors:** H. Siller

**Affiliations:** EIWH, Dun Laoghaire, Ireland

## Abstract

**Abstract:**

Intercultural psychotherapy aims at an understanding of and translating between cultural differences which may be experienced between the psychotherapist and the client. This is especially important when considering that due to crisis, conflict and war, but also due to globalisation and increased mobility, an increased number of clients with a migration background seek to undergo psychotherapy. In this presentation, current literature on needs and challenges of intercultural psychotherapy is discussed with regard to an intersectionality framework and more specifically in the context of power and privileges. These frameworks provide a useful understanding of cultural competency and sensitivity in intercultural psychotherapy.

**Disclosure of Interest:**

None Declared

